# Clinical-oriented 3D visualization and quantitative analysis of gingival thickness using convolutional neural networks and CBCT

**DOI:** 10.3389/fdmed.2025.1635155

**Published:** 2025-08-18

**Authors:** Lan Yang, ZiCheng Zhu, Yongshan Li, Jieying Huang, Xiaoli Wang, Haoran Zheng, Jiang Chen

**Affiliations:** ^1^ School of Stomatology, Craniomaxillofacial Implant Research Center, Fujian Medical University, Fuzhou, Fujian, China; ^2^ Guangdong Engineering Research Center of Oral Restoration and Reconstruction & Guangzhou Key Laboratory of Basic and Applied Research of Oral Regenerative Medicine, Department of Oral Implantology, School and Hospital of Stomatology, Guangzhou Medical University, Guangzhou, Guangdong, China; ^3^ School of Data Science & School of Science and Engineering, The Chinese University of Hong Kong, Shenzhen, China; ^4^ School of Intelligent Vehicles, Guangzhou Panyu Polytechnic, Guangzhou, China

**Keywords:** 3D visualization, gingival thickness, deep learning, cone beam computed tomography, implant restoration

## Abstract

**Objective:**

Traditional gingival thickness (GT) assessment methods provide only point measurements or simple classifications, lacking spatial distribution information. This study aimed to develop a CBCT-based 3D visualization system for gingival thickness using deep learning, providing a novel spatial assessment tool for implant surgery planning.

**Methods:**

CBCT and intraoral scanning (IOS) data from 50 patients with tooth loss were collected to establish a standardized dataset. DeepLabV3+ architecture was employed for semantic segmentation of gingival and bone tissues. A 3D visualization algorithm incorporating vertical scanning strategy, triangular mesh construction, and gradient color mapping was innovatively developed to transform 2D slices into continuous 3D surfaces.

**Results:**

The semantic segmentation model achieved a mIoU of 85.92 ± 0.43%. The 3D visualization system successfully constructed a comprehensive spatial distribution model of gingival thickness, clearly demonstrating GT variations from alveolar ridge to labial aspect through gradient coloration. The 3D model enabled millimeter-precision quantification, supporting multi-angle and multi-level GT assessment that overcame the limitations of traditional 2D measurements.

**Conclusion:**

This system represents a methodological advancement from qualitative to spatial quantitative GT assessment. The intuitive 3D visualization serves as an innovative preoperative tool that identifies high-risk areas and guides personalized surgical planning, enhancing predictability for aesthetic and complex implant cases.

## 1 Introduction

Gingival thickness (GT), which is regarded as an important component of the periodontal phenotype, plays pivotal roles in the health and function of teeth and dental implants (1). Besides, the phenotypes of periodontal soft tissues, including GT, are crucial for the outcomes of periodontal and implant therapy, orthodontic treatment as well as prosthetic rehabilitation (2). Accordingly, the assessment of such key component at all stages of treatment planning and execution is essential to achieve optimal clinical outcomes. Notably, a GT threshold of 1 mm was established to differentiate between thin gingiva (<1 mm) and thick gingiva (≥1 mm) at the 2017 World Symposium, while it also emphasized that thin gingiva can bring about greater risk of gingival recession (3).

The quality and stability of peri-implant soft tissues are influenced by multiple critical factors, including implant position, platform angulation, and gingival anatomical characteristics. In particular, GT has a direct impact not only on the aesthetic outcome but also on long-term survival of the implants. Thin gingival phenotype is closely associated with an increased risk of unfavorable surgical outcomes (4). Following tooth extraction, significant morphological and histological changes occur in the alveolar ridge. With the combination of the increased dimensional requirements of peri-implant mucosa compared to natural dentition and functions with fundamentally different biological attachment mechanisms, these alteration creates unique challenges for soft tissue management (5). Consequently, accurate assessment of gingival thickness becomes critically important in implant treatment planning, as it directly influences both aesthetic outcomes and long-term implant stability in this altered environment.

Over the years, various methods and tools have been developed and utilized to assess GT (6). Among the quantitative methods, transgingival probing (TGP), cone-beam computed tomography (CBCT) (6) and ultrasonography (US) (7) are widely used. Additionally, qualitative assessment of GT in clinical practice is performed using the probe transparency method, and the gingiva is classified as either thin or thick using a conventional periodontal probe (PPB) or a specially designed color-coded probe (CCP) (1).

CBCT scanning has been widely adopted for hard tissue imaging due to its superior diagnostic capabilities through three-dimensional reconstructed images. One disadvantage of CBCT imaging is its low soft tissue contrast resolution, which results from scatter radiation produced by structures outside the field of view (FOV) (8). This technical limitation makes it significantly difficult to accurately identify the gingival boundaries within CBCT datasets. To address this challenge, an approach utilizing CBCT in conjunction with a lip retractor has been proposed as an alternative methodology (9). Recently, a systematic evaluation was conducted to assess the consistency of various methods, including US, CBCT and TGP. The results demonstrated that there was a correlation between CBCT measurements and the histological gold standard, but the corresponding correlation of CBCT exhibited the lowest among all quantitative measurement methods (10, 11).

In recent years, the rapid development of intraoral scanning (IOS) technology has provided new possibilities for soft tissue assessment, and thereby high-precision digital reconstruction of the surface of teeth and soft tissues can be realized (12). Although CBCT can visualize internal structures, it is difficult to distinguish the boundaries of different soft tissues. Previous studies demonstrated that the combination of CBCT with IOS technology can provide a non-invasive and efficient method for soft tissue measurement, which can achieve a comprehensive assessment of periodontal phenotypes (13). However, this process requires lots of human resources, professional training, and complicated and time-consuming operations, which makes it difficult to be widely used in clinical practice. As an important branch of artificial intelligence (AI), deep learning has made significant breakthroughs in the field of medical image processing (14). Compared with conventional image processing methods, deep learning shows superiority in processing complex medical images, which can automatically learn feature representation through multi-layer neural networks rather than dependent on manually designed feature extractors (15). Especially in segmentation tasks, convolutional neural network (CNN) architectures such as U-Net have exhibited excellent performance in various medical image segmentation (16). It the area of oral imaging, deep learning has been successfully applied to clinical tasks such as tooth segmentation (17) and caries detection. Nevertheless, the practical applications in the precise identification of gingival soft tissues remain relatively limited.

Due to the blurred boundaries of soft tissues in CBCT images, traditional manual or semi-automatic segmentation methods are usually inefficient and easily affected by subjective factors. In contrast, deep learning has the capabilities of powerful feature extraction and pixel-level classification, which is expected to overcome this technical difficulty and provide a promising strategy for accurate segmentation of soft tissues in CBCT. Meanwhile, some existing studies have demonstrated the potential application value of deep learning in soft tissue segmentation. For example, a Dense U-Net model achieving 93.43% accuracy in segmenting masseter and tongue muscles in head MRI images through automatic feature representation learning via multi-layer neural networks rather than relying on manually designed feature extractors (18). Addtionally, a novel AI-based method was proposed for gingival segmentation, but its applicability is limited as it was only tested in an animal pilot study using Yorkshire pig mandibles (19), and thereby further validation in human subjects is still required to assess its clinical generalizability.

To address these limitations, a deep learning-based method is designed for precise gingival segmentation in human CBCT images. As the intraoral scanner (IOS) data is used as the gold standard, the proposed approach can improve the accuracy and repeatability of gingival thickness (GT) measurements while reducing the manual workload. Additionally, an innovative gingival thickness visualization method is also developed to transform the irregular curved gingival surface into a quantifiable three-dimensional plane, which can provide the clinicians with pre-implantation assessment and surgical planning.

## 2 Materials and methods

### 2.1 Design of experiment

This retrospective study included 50 patients with edentulous teeth who received treatment at the Department of Implant Dentistry, Affiliated Stomatological Hospital of Guangzhou Medical University within the period from January 2023 to December 2023. The study protocol was approved by the Ethics Committee of the Affiliated Stomatological Hospital of Guangzhou Medical University (Approval No.: LCYJ20250303004), and due to the retrospective nature of this study, the requirement for written informed consent was waived. This study was carried out in strict accordance with the ethical principles outlined in the Declaration of Helsinki (2024 version) issued by the World Medical Association.

**Figure 1:**
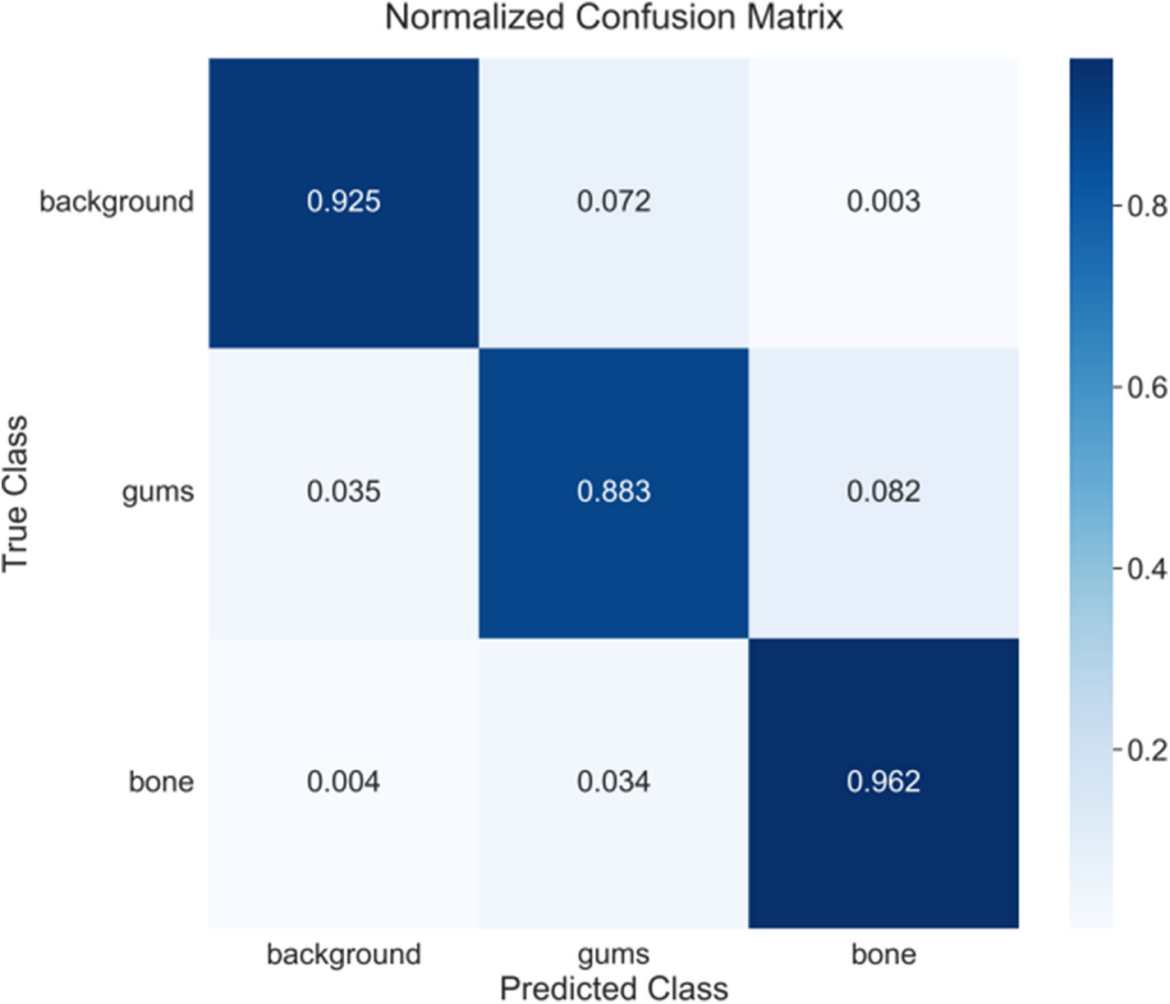
Schematic diagram of research design process.

The inclusion criteria for this study are listed as follows: (1) age within the range of 18–65 years; (2) requirement of implant restoration treatment; (3) availability of complete preoperative CBCT and IOS data. Exclusion criteria are also listed as follows: (1) suffering from severe systemic diseases; (2) poor oral hygiene; (3) presence of obvious artifacts or distortions in CBCT or IOS data; (4) suffering from severe periodontal disease. Figure 1 illustrates the schematic diagram of research design process.

### 2.2 Data acquisition

Preoperative CBCT and digital IOS data were collected from the patients. CBCT images were acquired using a Planmeca ProMax 3D scanner (Planmeca Oy, Helsinki, Finland). All CBCT images were acquired following the institution's standardized protocol. This protocol stipulated the use of cotton rolls to separate the lip and cheek soft tissues from the gingiva, aiming to prevent their overlap and thus ensure optimal image quality for periodontal assessment. CBCT data were saved in DICOM format. The scanning parameters were set as follows: voltage of 90 kV, current of 4 mA, scan time of 28 s, voxel size of 0.3 mm^3^, and field of view (FOV) of 70 mm × 130 mm. Digital IOS was conducted using a 3Shape TRIOS intraoral scanner (3Shape A/S, Copenhagen, Denmark), and the acquired data were exported in STL format for subsequent analysis.

### 2.3 Dataset construction

#### 2.3.1 High-precision fusion of oral optical scanning and CBCT

In this work, an innovative method was adopted through converting the three-dimensional feature point detection issue into a two-dimensional one to achieve the high-precision fusion of CBCT and oral optical scanning data. The specific implementation steps can be described as follows. First, two accurate three-dimensional surface models were generated from two imaging modalities of CBCT and oral optical scanning, respectively. Subsequently, a series of two-dimensional projection images was produced with an optimized angle interval od 2*π*/9. Given the specific characteristics of oral structures, a feature point extraction algorithm was employed to process these projection images and identify key anatomical landmarks in the oral cavity. The point extraction algorithm combined scale-invariant feature transform (SIFT) and the Harris corner detector, and the RANSAC algorithm was used for feature matching and screening to accurately map these two-dimensional feature points back to the three-dimensional coordinate system. Besides, 55 potential dentition feature points were strictly evaluated and 10 anatomical positions with the highest geometric stability under different imaging environments were selected as registration reference points. After calculating the initial rigid transformation matrix on the basis of these feature points, the iterative closest point (ICP) algorithm was used to refine the registration on the local tooth surface area containing the feature points, and then high-precision spatial registration of CBCT and oral scan data can be obtained.

#### 2.3.2 Data annotation method

After completing the registration, the external gingival contour from the oral optical scanning was accurately projected into the CBCT to generate the external boundary contour of the gingiva. The dental arch curve in the cross-sectional image was selected to obtain a dental arch cross-sectional image. The dental arch cross-sectional image was cropped to extract the region of interest containing the external boundary contour, which were used as the raw data for annotation. After then, the clinical experts manually annotated the tooth area to create an alveolar bone mask. The outer boundary of alveolar bone mask formed the boundary between the alveolar bone and the gingiva. The region enclosed between the boundary line and the previously projected gingival external contour line was defined as the gingival mask. Eventually, a PNG image was exported in VOC format, and different pixel values (1, 2, 0) were assigned to the tooth area, gingival area and background, respectively.

#### 2.3.3 Dataset construction

Based on the differences in the edentulous area, 20–50 cross-sectional images were obtained from each patient. After the high-precision fusion of CBCT and oral optical scanning of each patient, a total of 1,435 data samples were collected using the above data annotation method in Section 2.3.2. To ensure the quality and consistency of the dataset, a systematic preprocessing process was conducted for all cross-sectional images. First, all images were adjusted to the resolution of 512 × 512 pixels to ensure the standardization of input data dimensions. Subsequently, the intensity normalization technique was used on the basis of window width and window position adjustment to map the pixel values of image within the range of [0,1], and thereby the contrast of tooth-gingival interface was optimized. To inhibit the specific low-frequency noise of CBCT, adaptive histogram equalization was utilized to enhance the clarity of tissue boundaries, and bilateral filtering technology was integrated to retain the key anatomical boundaries while decrease the noise interference. Eventually, in order to promote the generalization ability of model, data augmentation strategies were performed to effectively expand the diversity of training samples, including random rotation (±15°), horizontal flipping, and slight brightness/contrast adjustment (±10%). The training set, validation set, and test set were divided in a ratio of 7:1:2 to ensure the strict separation of data from different patients, and then the data leakage that can affect the objectivity of model evaluation was avoided.

### 2.4 Establishment of deep learning model

#### 2.4.1 Network architecture

In this study, DeepLabV3+ (20) was adopted as the basic architecture for semantic segmentation, and the network can effectively extract the semantic information from the images while maintaining high computational efficiency. The network architecture was primarily composed of encoder and decoder. The encoder used MobileNetV2 (21) as the backbone network, while the decoder was used to fuse multi-scale features. Particularly, the encoder used MobileNetV2 as the backbone network, which was a lightweight deep CNN that utilized deep separable convolution to significantly reduce the computational parameters while maintaining a high feature extraction capability. During the feature extraction process, the network first obtained the features at two scales, i.e., shallow features with the dimensions of [128, 128, 24] to retain detail information, and backbone features with the dimensions of [30, 30, 320] to extract high-level semantic information.

The core component of network is Atrous Spatial Pyramid Pooling (ASPP) module, which used dilated convolutions with different dilation rates to extract multi-scale contextual information. The ASPP module was composed of five parallel branches, including 1 × 1 standard convolution, 3 × 3 dilated convolutions (dilation rates of 6, 12, and 18, respectively), and a global average pooling branch. These branches captured contextual information at various scales and then fused them to provide a relatively comprehensive feature representation. In the decoder part, the network upsampled the output of ASPP module and fused it with the low-level features from the encoder. Such design can effectively integrate the high-level semantic information and low-level spatial details, leading to the accurate segmentation results. Eventually, the features were mapped to the number of target categories via 1 × 1 convolution and upsampled to the original input resolution to generate the final segmentation map.

#### 2.4.2 Loss function

In present work, a composite loss function was used to optimize network training. The combination of cross-entropy loss and Dice loss was adopted to address the sample imbalance issue between different categories.

The cross-entropy loss function can be defined as follows [Equation (1)]:

(1) $$L_{\mathrm{CE}}=-\sum_{c=1}^{C}y_{c}\log\;(\;p_{c})$$

where $y_{c}$ is the true label of category *c*, $p_{c}$ is the probability predicted by the network for the category.

The Dice loss (22) can be expressed on the basis of the Dice coefficient [Equation (2)], which was used to measure the overlap between the predicted segmentation and the truth segmentation.

(2) $$L_{\mathrm{Dice}}=1-\frac{2\sum_{i}^{N}\;p_{i}y_{i}}{\sum_{i}^{N}\;p_{i}^{2}+\sum_{i}^{N}y_{i}^{2}}\;$$

where $p_{i}$ is the predicted probability, $y_{i}$ is the true label.

The total loss function [Equation (3)] can be described as follows:

(3) $$L_{\mathrm{total}}=\alpha L_{\mathrm{CE}}+\beta L_{\mathrm{Dice}}\;$$

where *α* and *β* are the weighting coefficients that balance the two loss components.

Additionally, to further address the sample imbalance issue between different categories, the category weights were obtained by assigning different loss weights to each category, which allows the network to pay attention to the categories with fewer samples.

#### 2.4.3 Training parameters

The training strategy followed a staged approach, including a freezing training phase and an unfreezing training phase. In the freezing training phase, the parameters of backbone network were frozen, and only the other parts were trained, which was favorable for preventing the pre-trained weights from excessive modification during the initial phase. Stochastic gradient descent (SGD) (23) was used as the optimizer. In order to avoid overfitting, a momentum parameter was set as 0.9 and a weight decay was set as 1 × 10^−4^. The initial learning rate was set as 7 × 10^−3^, while the minimum learning rate was 0.01 times the initial learning rate, and the learning rate decay followed a cosine annealing strategy. During the freezing training phase, the batch size was 8, whereas during the unfreezing training phase, the batch size was reduced to 4 to accommodate the relatively complex training. The freezing training phase was conducted for 50 epochs, and the total number of training epochs reached 200. The downsampling factor of network was set as 16 to balance segmentation accuracy and computational efficiency. The learning rate was adjusted using an adaptive strategy on the basis of the batch size [Equation (4)].

(4) $$lr_{\mathrm{adjusted}}=\min(\max(\mathrm{batch}\_\mathrm{size}/nbs\times lr_{\mathrm{init}},lr_{\min}),lr_{\max})$$

where nbs is the reference batch size of 16, $lr_{\mathrm{init}}$ is the initial learning rate, $lr_{\min}$ and $lr_{\max}$ are the lower and upper limits of learning rate, respectively.

### 2.5 Evaluation metrics

Aiming at comprehensively assessing the performance of model, a variety of evaluation metrics were applied. The Mean Intersection over Union (mIoU) was the primary evaluation metric, and thereby the IoU value of each category was calculated average. IoU was identified as follows.

(5) $$\mathrm{IoU}=\frac{\mathrm{TP}}{\mathrm{TP}+\mathrm{FP}+\mathrm{FN}}$$

where TP, FP and FN represent true positives, false positives, and false negatives, respectively.

Meanwhile, the Precision was used to evaluate the accuracy of model [Equation (6)].

(6) $$\mathrm{Precision}=\frac{\mathrm{TP}}{\mathrm{TP}+\mathrm{FP}}$$

The Recall was used to evaluate the completeness of model [Equation (7)].

(7) $$\mathrm{Recall}=\frac{\mathrm{TP}}{\mathrm{TP}+\mathrm{FN}}$$

Besides, the F1 score [Equation (8)] was calculated, which was the harmonic mean of Precision and Recall.

(8) $$F1=\frac{2\times \mathrm{Precision}\times \mathrm{Recall}}{\mathrm{Precision}+\mathrm{Recall}}$$

The performance of model was evaluated on the validation set every 5 training rounds to monitor the training progress and avoid overfitting. This evaluation was conducted with the same input size and batch size to ensure the consistency and comparability of results, and the evaluation results were also recorded in the loss history for subsequent analysis and visualization.

### 2.6 Gt measurement and visualization workflow

#### 2.6.1 Image processing method

The image processing workflow started with the color analysis of the semantic segmentation resultant image, and the key structures in the oral cavity were identified by separating the BGR (blue-green-red) color channels. As the gingival tissue appeared red, the tooth surface was pre-marked as green, and the precise color thresholds were adopted (red area: R > 75, G < 30, B < 30; green area: G > 75, R < 30, B < 30) to create binary masks corresponding to these two tissues, respectively. Subsequently, the green mask was converted to an 8-bit single-channel image, and the morphological closing operation was employed to smooth edges and fill small holes. The contours can be detected, while the largest area was selected as the main boundary of tooth surface. The processing process excluded the boundary points with y coordinate of 0 and resolved the problem of duplicate *x* coordinate, in which the points with the smallest *y* value was retained). Finally, all valid contour points were sorted by *x* coordinate to generated a continuous boundary line. During the thickness measurement process, each position along the image width was scanned pixel by pixel. For each *x* coordinate, the nearest boundary point was identified as the starting position, and then the contiguous red pixels were counted along the specified direction (upward or downward). The measurement process followed the intelligent decision logic: once entering the red area, it continued to count until it exited, thereby accurately capturing the complete GT. In order to reduce measurement noise and random fluctuations, the raw thickness data was processed by one-dimensional Gaussian filter, and the degree of smoothing was controlled by the adjustable sigma parameter.

After processing, the system annotated the green contour points (represented by small yellow dots) on the original image, and vertical lines were drawn to determine the GT at each location. Meanwhile, a thickness distribution graph was used to demonstrate the lateral variation of GT. All results were saved as high-resolution images for subsequent analysis.

#### 2.6.2 Data processing and integration

During the data processing, a single image was first processed. The GT value was extracted from each *x* coordinate position, and the corresponding slice number was recorded. These raw measurement data were organized into a structured table, which contained key information such as image name, slice number, *x* coordinate, GT as well as measurement direction. When multiple images were processed, the resultant slices were sorted in natural order based on the filenames to ensure the correct slice sequence, and then the data of all individual images were merged into a unified dataset, which can comprehensively reflect the three-dimensional thickness distribution of the entire gingival area. The merged dataset was saved in CSV format.

With regards to visualization, the system generated various figures to present the data features at different levels: the thickness distribution scatter plot showing the relationship between the slice number, *x* coordinate and thickness within the entire dataset, and thereby the color of the points was mapped to the GT for the formation of an intuitive heatmap effect.

## 3 Results

### 3.1 Performance of deep learning models

**Figure 2:**
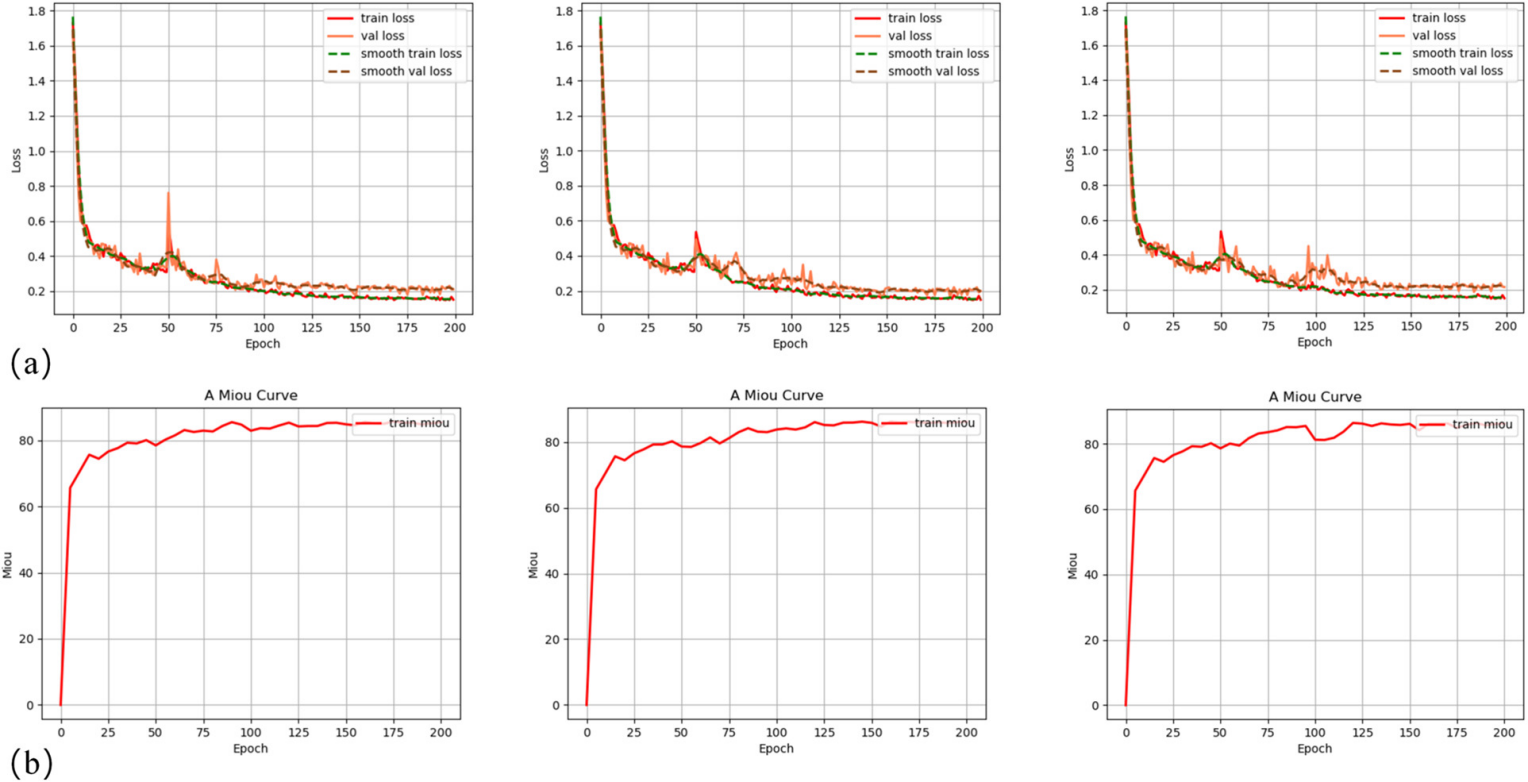
Training curves ofDeepLabV3+ model: **(a)** training and validation loss curves for three experimental groups; **(b)** mean Intersection over Union (mIoU) curves on the training dataset for three experimental groups.

In order to clarify the stability of DeeplabV3+ model training and the reproducibility of results, three repeated model training experimental results demonstrate similar trends in loss reduction and mIoU increase (Figure 2). It can be found that the DeepLabV3+ model exhibits excellent performance in oral tissue segmentation with an average IoU of 85.92 ± 0.43% (Table 1). It should be noted that the model can perform well in bone tissue segmentation (mIoU of 89.83 ± 0.28%, average recall of 96.06 ± 0.32%), while the gingival segmentation shows inferior performance (IoU of 76.99 ± 0.66%) but it still maintains good accuracy. Such discrepancy may reflect that bone tissue has clearer boundary features in the image, whereas the gingiva has a lower contrast with surrounding soft tissues, leading to the increased difficulty of segmentation. The high accuracy of model on the background category (97.19 ± 0.38%) can further verify its robustness in complex oral environments.

**Table 1:** Segmentation performance of DeeplabV3+.

Performance	Mean	Gingiva	Bone	Background
IoU	85.92 ± 0.43	76.99 ± 0.66	89.83 ± 0.28	90.95 ± 0.54
Recall	92.56 ± 0.20	88.21 ± 0.39	96.06 ± 0.32	93.41 ± 0.89
Precicion	92.87 ± 0.26	85.82 ± 0.98	93.26 ± 0.23	97.19 ± 0.38

**Figure 3:**
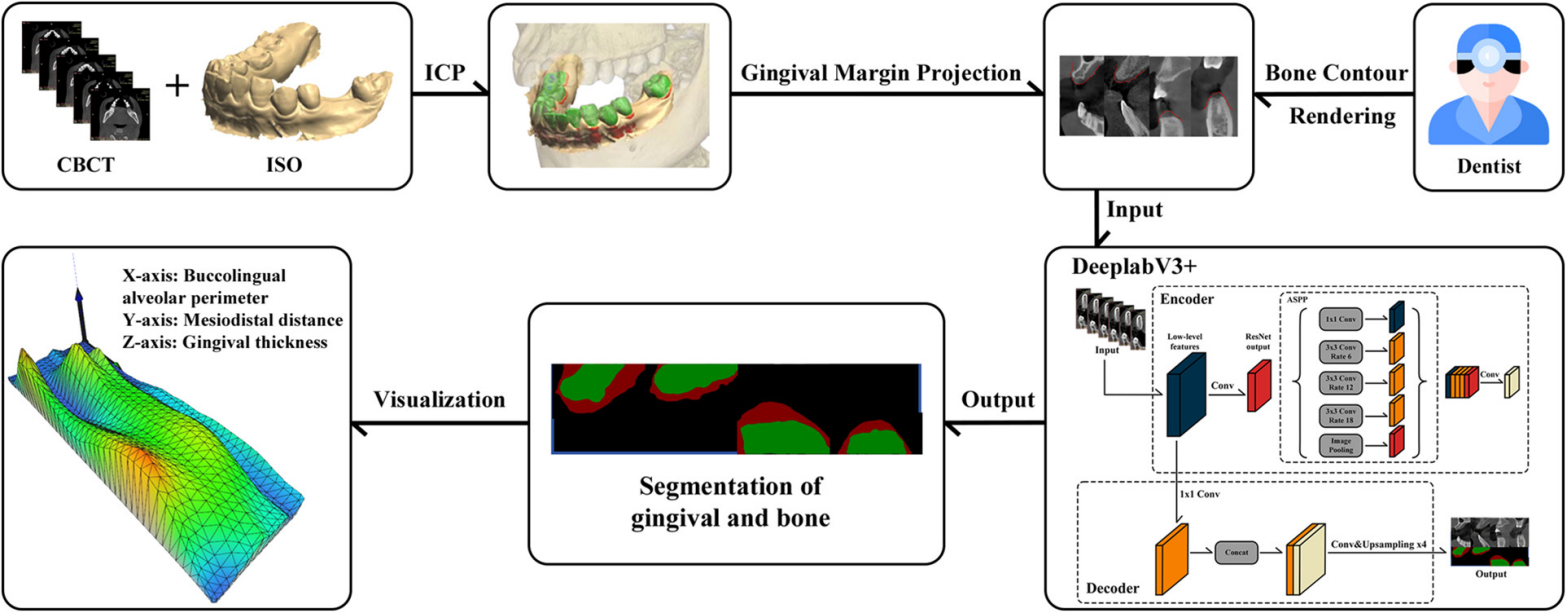
Segmentation results using DeepLabV3+ model: **(a)** original CBCT cross-sectional image of the dental arch; **(b)** segmentation results using DeepLabV3+, where red color represents gingiva and green color represents alveolar bone.

Figure 3 illustrates the segmentation results of CBCT scans. The upper row shows the original CBCT image slices, and the lower row shows the corresponding segmentation results. The red area represents the gingival, and the green area represents the bone tissue. It is apparent that in some CBCT images, the gingival and lip soft tissues are difficult to be directly distinguished. The deep learning-based segmentation algorithm can still effectively identify these structures, which may be achieved by learning the complex spatial correlations and features of tissue boundary. Nevertheless, due to the intrinsic black box feature of deep learning model, it is still difficult to fully elaborate the exact segmentation mechanism.

**Figure 4:**
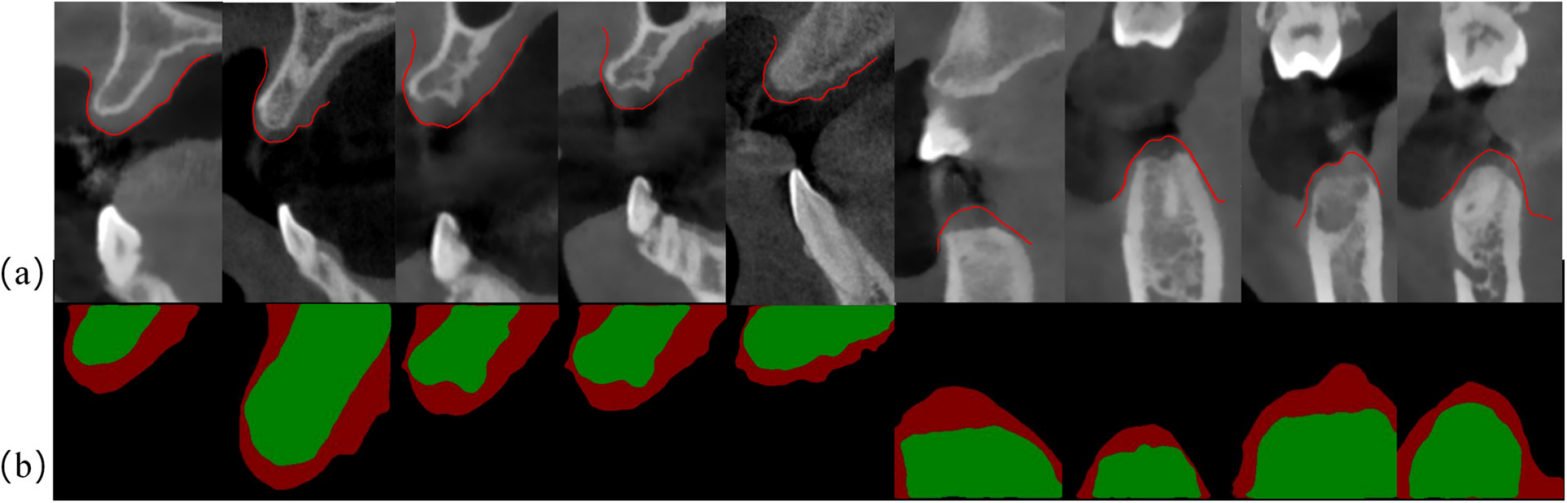
Confusion matrix of DeepLabV3+ model.

Figure 4 presents the confusion matrix of model with the best performance. After confirming the stability of method, the best results can be achieved for detailed analysis. The confusion matrix analysis (Figure 4) further validates the classification performance of model. In the background category, a correct classification rate reaches 92.5% with only 7.2% being misclassified as gingiva and 0.3% being misclassified as alveolar bone. The performance of gingiva segmentation is also highly reliable, reaching an accuracy of 88.3% with 3.5% being misclassified as background and 8.2% being misclassified as alveolar bone. The alveolar bone segmentation exhibits the highest accuracy of 96.2% with only 0.4% and 3.4% being misclassified as background and gingiva, respectively, which is consistent with the IoU evaluation, indicating that the model has excellent performance in bone tissue recognition. Besides, it also reflects the relative challenge in distinguishing the gingiva from other tissues (especially bone tissue).

### 3.2 Measurement and visualization of GT

**Figure 5:**
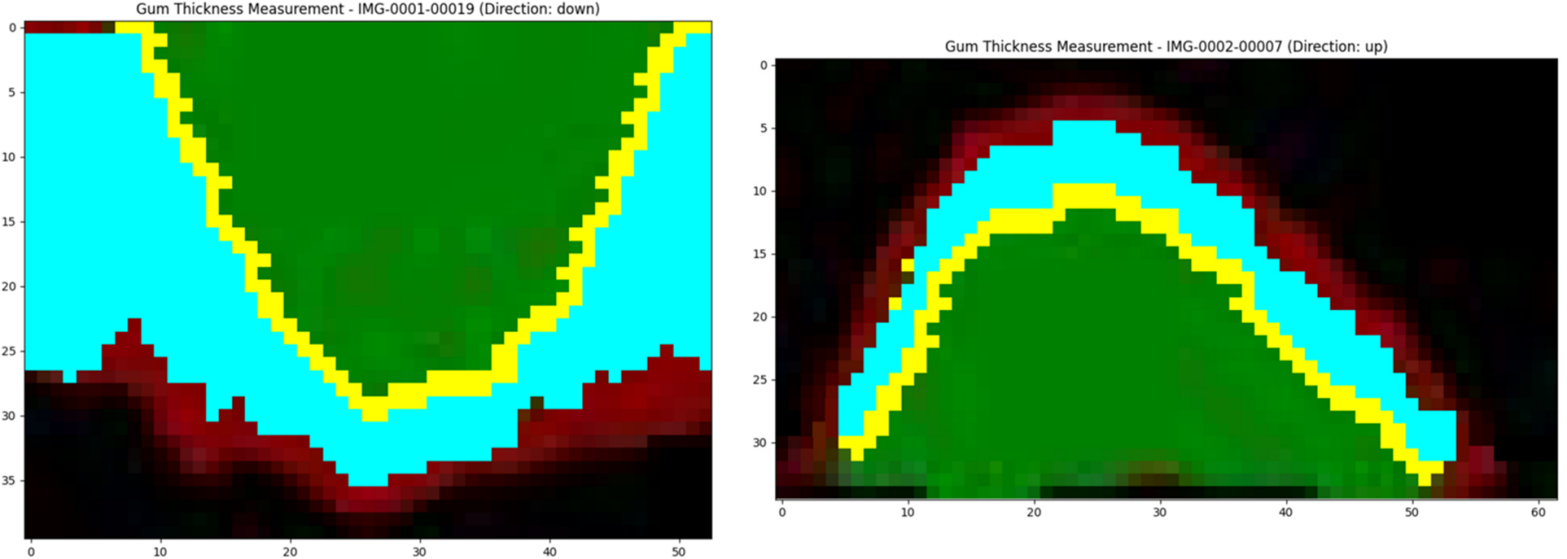
Gt calculation method.

Figure 5 presents the calculation results of GT through the slice case. These results were obtained using the method described in Section 2.7.2 and visualized using Matplotlib. As shown in Figure 5, it is evident that the yellow points mark the boundary contour line between the gingiva and the tooth surface, which is the key reference line extracted from the edge of green area (tooth surface). Based on this contour, the program performs thickness measurement in the direction of the external contour of gingiva at each x-coordinate position, and the blue area represents the valid calculation range of GT. The algorithm begins with the contour points and then intelligently tracks the continuous red pixels (GT) until the non-gingival area can be detected, and thus the GT values are accurately quantified at different positions. After smoothing by Gaussian filtering, the measured data exhibit the characteristics of smooth transition, and random fluctuations at the edge is eliminated. The yellow points accurately mark the boundary contour line between the gingiva and the tooth surface (green area). The algorithm measures the GT from these contour points, and then the light blue effective calculation area is generated. It is obvious that the GT on both sides of the tooth is slightly different. The gingiva in the central area is thinner than that in the edge area. The dark red area at the bottom represents the GT, which clearly shows its boundary with the tooth. Based on this thickness information, the thickness results of all CBCT slices of patient are integrated for further three-dimensional reconstruction.

**Figure 6:**
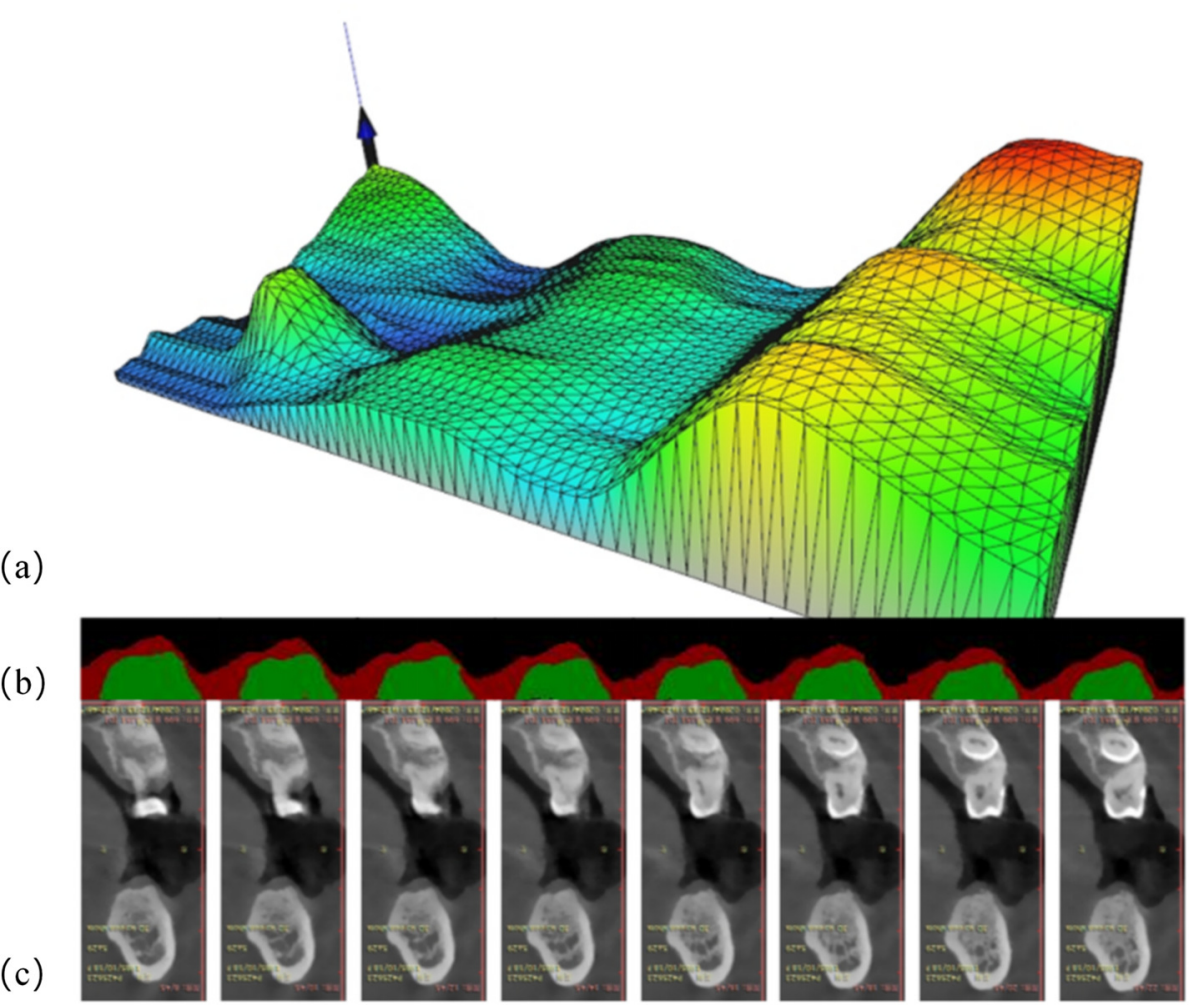
Three-dimensional visualization analysis of GT: **(a)** three-dimensional model exhibition: the *X*-axis represents the alveolar bone circumference from the buccal to the lingual sides, the *Y*-axis represents the mesiodistal distance from the patient's left to right side, and the *Z*-axis reflects the GT values. The color gradient represents the variations in issue thickness. **(b)** Segmentation results of consecutive cross-sectional slices using DeeplabV3+ deep learning algorithm: red and green markings show the identified boundaries of tissues. **(c)** Corresponding original CT cross-sectional scan images, showing the alveolar bone and surrounding soft tissue structures.

Figure 6 illustrates the complete workflow and effectiveness of three-dimensional reconstruction of GT. As shown in Figure 6a, the three-dimensional reconstruction results demonstrate the spatial distribution characteristics of GT, and a gradient color mapping from blue (thinner area) to green, yellow and red (thicker area) is used to map the variations in thickness. The three-dimensional surface shows obvious anatomical features, suggesting that the GT is varied regularly at different tooth positions. Specifically, the right area exhibits overall yellow-red tones, indicating that the palatal GT is larger. The central area is mainly green, indicating moderate thickness, while blue color appears from the alveolar ridge to the labial side, representing relatively thin GT. The mesh structure of model surface clearly displays the continuous surface generated by the triangulation algorithm, and thereby the subtle changes and transition areas of GT are successfully captured. The present three-dimensional visualization technology overcomes the limitations of traditional two-dimensional measurement, which can provide clinicians with a powerful tool for comprehensive assessment of gingival phenotype. It converts the gingival phenotype from overall consideration at the patient level to quantitative evaluation at the millimeter level. This method can not only guide the formulation of personalized treatment plans, but also holds important value in enhancing the aesthetic restoration effects and reducing the postoperative complications. Notably, it can provide clinicians with additional risk warnings during the actual execution of operation, such as the position that is more prone to perforation during flap elevation, the selection of tension-relieving sites after bone grafting and the mucosal relaxation.

## 4 Discussion

In this study, an innovative deep learning method was proposed for CBCT to evaluate GT and three-dimensional morphology in edentulous areas. By integrating the gingival surface morphology data captured by IOS technology and the bone edge contours precisely marked by clinicians on CBCT images, a comprehensive three-dimensional model of gingival soft tissue in the edentulous area was successfully established. The DeeplabV3 + deep learning algorithm was used to achieve high-precision segmentation of gingival tissue, and a special GT calculation method and innovative visualization algorithm were developed to obtain an intuitive three-dimensional visualization of GT.

Generally, the conventional gingival assessment methods mainly focus on simple qualitative classification of gingival phenotypes (such as thin or thick), which is difficult to meet the demands of contemporary precise dental treatment (24). This is critical in implant surgeries, especially when complex bone grafting procedures are involved, the thickness of each specific position of gingiva has significant impacts on the design of surgical plan (25). For instance, the difference in GT between the alveolar ridge crest and the buccal side may determine the selection of incision site, whereas the GT at the membrane-gingival junction can affect the selection of relaxation and tension-reducing approaches. Traditional methods cannot provide such spatially precise thickness distribution information, resulting in clinicians often relying on experience-based judgment, and the surgical risks and uncertainties are significantly increased. As reported, the integration of CBCT with IOS has represented a significant advancement in non-invasive periodontal assessment, enabling precise measurement of palatal mucosal thickness (26). It can be found that digital technologies have promote the comprehensive analysis of the periodontal phenotype in the maxillary anterior region (27), which can provide detailed evaluations of GT, bone thickness and keratinized gingival width with strong aesthetic correlations. However, despite these advancements, limitations remain, including variability in soft tissue imaging accuracy depending on operator expertise, potential image distortion in CBCT, and limited accessibility or cost-effectiveness in routine clinical settings. Therefore, in present work, the proposed three-dimensional assessment system has achieved the first accurate quantification and visualization of GT in the entire edentulous area, allowing clinicians to formulate more precise surgical strategies according to the individual GT distribution characteristics of EACH patient. Especially in the aesthetic area and complex cases, this precise assessment can significantly improve the predictability and aesthetic effect of surgery.

Compared to previous pilot animal study, the present work demonstrates significant improvement in both technical methodology and clinical application. Meanwhile, a U-Net architecture was used to validate the feasibility of AI-assisted gingival assessment in a limited sample of porcine models, and then a correlation coefficient between virtual and clinical measurements can be determined as of *r* = 0.9656 and a mean difference reached 0.066 ± 0.223 mm (19). In present work, the DeepLabV3+ (28) architecture with its Atrous Spatial Pyramid Pooling (ASPP) module is utilized to achieve more precise segmentation of the complex anatomical boundaries in the human oral cavity. It should be noted that the average accuracy achieved in this study can be determined 92.87 ± 0.26%, which is slightly lower than the result reported in the existing literature. This is derived from fundamental differences in evaluation methodology. In the present study, a comprehensive pixel-wise assessment strategy is adopted to obtain fine-grained quantification across the entire image region, whereas only 43 preselected discrete measurement points were assessed in previous study (19, 28). These points are usually selected at anatomical locations with clear boundaries and easy identification, and thereby the inertial bias is inevitably introduced. In terms of image fusion, the semi-automated CloudCompare software was employed to align CBCT with intraoral scans (29). In contrast, a novel approach is designed to transform the three-dimensional feature point detection into a two-dimensional issue. With the combination of Scale-Invariant Feature Transform (SIFT) and the Harris corner detector, the method can select the 10 anatomical positions with the highest geometric stability from 55 potential dental feature points as alignment reference points, leading to the improved spatial alignment accuracy.

The DeepLabV3+ architecture used in this work demonstrate outstanding performance in the semantic segmentation task of oral images. Based on MobileNetV2, the lightweight backbone network can effectively balance the computing resource requirements and segmentation accuracy, enabling the system to operate efficiently on standard computing devices. The ASPP module establish multi-scale perception capabilities using dilated convolution with various dilation rates, which is essential for oral tissue recognition, as the boundary between gingiva and teeth has scale diversity and complex morphological changes. The experimental results demonstrate that the algorithm can accurately distinguish between gingival tissue and tooth surface, and thereby high stability can be maintained even under blurred tissue boundaries, which is of great significance in clinical applications (30).

The introduction of the combined loss function can effectively address the issue of category imbalance in oral images, while the unbalanced proportion of gingiva and teeth in the image is often detected, but it is difficult for the traditional single loss function to simultaneously take into account both accuracy and recall rate. The proposed training strategy following a staged approach can further facilitate the performance of model. In the freezing training phase, the pre-training weights are protected, and in the unfreezing training phase, the parameters of network are fine-tuned to avoid overfitting while maximizing the capability of model to extract oral features. The above evaluation metrics show that the algorithm can obtain high segmentation accuracy on the test dataset, which provides a solid foundation for subsequent GT measurements.

The proposed GT technology begins with color analysis, and precise color thresholding is applied to separate the gingival and tooth tissues. Meanwhile, with the combination of morphological processing technology, tissue boundaries can be identified. This strategy fully utilizes the inherent features of oral tissues to improve measurement accuracy. Unlike the existing simple image-based algorithms (31), this novel method integrates multi-level image processing technology to realize a fully automated transformation from raw image to precise thickness values. The core innovation of algorithm can be ascribed to its vertical scanning strategy and intelligent judgment logic, which conducts pixel-by-pixel analysis of the variation of GT along tooth surface. This logic specifically designs an accurate method for gingival area recognition, i.e., continuously scanning along the predetermined direction from the starting point of the tooth-gingival boundary, intelligently detecting the entry and exit points of gingival area, and precisely capturing the complete thickness. Compared with traditional methods, this pixel-level measurement approach with the resolution of 0.1 mm can capture subtle variation in GT, which can provide more detailed information for clinical evaluation. Furthermore, the introduction of Gaussian filtering can effectively reduce measurement noise and random fluctuations, ensuring the continuity and reliability of thickness data while retaining clinically relevant thickness change patterns. In contrast to commercial US thickness measurement, this algorithm can theoretically achieve single-pixel level measurement precision, and thus the accuracy is remarkably enhanced.

It should be noted that the algorithm resolves a variety of technical issues. First, it addresses the issue of boundary discontinuity through the morphological closing operations to smooth edges and fill small holes. Moreover, these technical innovations enable the algorithm to handle various complex oral anatomical structures, which provide reliable measurements even in cases of blurred boundaries, uneven lighting or abnormal gingival morphology, indicating strong adaptability and robustness. Nevertheless, merely obtaining GT data is insufficient to effectively guide clinical decision-making. In fact, converting the complex three-dimensional thickness data into intuitive and comprehensible visual representation is essential for the value of clinical applications. The multi-level visualization method developed in this study can provide robust support for the interpretation and application of GT data. In single-image visualization, boundary point annotations and vertical lines show the intuitive depiction of the measurement process, allowing clinicians to directly evaluate the variation in thickness on the original image. Additionally, the thickness distribution map can provide a global view of lateral changes to identify the potential abnormal areas.

Besides, the visualization method of multi-image data integration is particularly innovative. The three-dimensional thickness distribution scatter plot integrates the slice number, *x* coordinate and thickness into an intuitive heat map, and the visualization of the three-dimensional structure of the entire gingival area can be achieved, which holds significant value in clinical evaluation. The single-slice line graph and multi-slice comparison graph demonstrate the thickness change pattern in different anatomical positions, allowing clinicians to quickly identify abnormalities in specific areas or conduct comparative analysis between different sites. These visualization tools not only improve the efficiency of data interpretation, but also promote the accuracy of clinical decision-making. The calculation and output of statistical summaries further enhance the possibility of quantitative analysis, which provides an objective basis for evidence-based diagnosis and treatment plans. This multi-dimensional and multi-scale visualization method can convert complex measurement data into intuitive visual representations and significantly improve the feasibility of clinical applications, which also provides rich data basis for subsequent study.

Despite the remarkable achievements of this study, there are still some limitations. The existing thickness measurement is mainly based on two-dimensional images, which may not fully capture the three-dimensional morphology of gingiva. Accordingly, the combination of three-dimensional reconstruction technology will be an important direction for the future development, and comprehensive three-dimensional data can be achieved via CT or optical scanning. Meanwhile, although the DeepLabV3+ architecture shows excellent performance, it still has limitations in capturing the long-range dependencies of complex oral anatomical structures. The introduction of Transformer-based segmentation algorithms (such as SegFormer) will be an important technical breakthrough in the future. Owing to its self-attention mechanism, the Transformer can effectively establish relationships between long-distance pixels in the image, which is important for understanding the overall structure of oral tissues. Especially for subtle structures such as the gingival margin, the Transformer architecture may provide more accurate segmentation results. Additionally, the hybrid models can reduce computational overhead while maintaining accuracy, which makes it suitable for real-time applications in clinical environments.

In terms of three-dimensional reconstruction, the two-dimensional thickness analysis in this study can provide valuable information, but it is insufficient to fully characterize the three-dimensional morphology of gingiva. The development of dedicated three-dimensional surface reconstruction tools will remarkably enhance the clinical value of data. Specifically, a continuous three-dimensional surface can be constructed by integrating adjacent slice data, and a direct triangulation method can be unitized to create connecting surfaces between slices. Meanwhile, Laplace smoothing algorithm can be applied to generate smooth natural surfaces. The comprehensive three-dimensional reconstruction process should include a few steps such as preprocessing, triangulated mesh construction, surface smoothing, and color mapping, and eventually an interactive three-dimensional model is established, which allows clinicians to observe the gingival morphology and thickness distribution from any angle.

A key future direction is to advance from single-tissue segmentation to the differentiation of keratinized gingiva from alveolar mucosa, a distinction critical for comprehensive periodontal phenotype assessment and surgical planning. Given the challenge of low contrast resolution between these tissues on CBCT, we propose developing a multi-class semantic segmentation model that leverages a multi-modal data fusion strategy. This approach will integrate CBCT's internal anatomical data with the high-resolution surface color and texture from IOS. Such fusion is expected to enable precise identification of the mucogingival junction, evolving our system into a comprehensive tool for soft tissue phenotype analysis and enhancing predictability in advanced mucogingival surgeries.

## 5 Conclusion

In this study, a deep learning-based three-dimensional gingival thickness assessment system for edentulous areas was successfully developed. With the combination of the intraoral scanning (IOS) cone-beam computed tomography (CBCT) data, and the DeeplabV3+ segmentation algorithm, a methodological breakthrough can be achieved, which realized the transition from traditional qualitative classification of gingival phenotype to position-specific quantitative assessment. As expected, the precise three-dimensional visualization model of gingival thickness produced by this system can provide an intuitive preoperative assessment tool for clinicians.

## Data Availability

The datasets presented in this article are not readily available because The raw/processed data supporting this study are not publicly available due to confidentiality agreements with research participants and ethical restrictions imposed by the institutional review board. Interested researchers may submit requests for de-identified data to the corresponding author, subject to approval and data sharing agreement. Request to access the datasets should be directed to Haoran Zheng, zheng980722@gmail.com.

## References

[1] De Rouck T Eghbali R Collys K De Bruyn H Cosyn J. The gingival biotype revisited: transparency of the periodontal probe through the gingival margin as a method to discriminate thin from thick gingiva. J Clinic Periodontol. (2009) 36:428–33. 10.1111/j.1600-051x.2009.01398.x 19419444

[2] Aşkın Dİ Tayman MA Çelik B Kamburoğlu K Özen D. Comparison of gingival and periodontal phenotype classification methods and phenotype-related clinical parameters: cross-sectional observational study. BMC Oral Health. (2025) 25:620. 10.1186/s12903-025-06007-0 40269862 PMC12020244

[3] Jepsen S Caton JG Albandar JM Bissada NF Bouchard P Cortellini P Periodontal manifestations of systemic diseases and developmental and acquired conditions: consensus report of workgroup 3 of the 2017 world workshop on the classification of periodontal and peri-implant diseases and conditions. J Clin Periodontol. (2018) 45::S219–29. 10.1111/jcpe.12951 29926500

[4] Kim DM Bassir SH Nguyen TT. Effect of gingival phenotype on the maintenance of periodontal health: an American academy of periodontology best evidence review. J Periodontol. (2020) 91:311–38. 10.1002/jper.19-0337 31691970

[5] Won C. A novel framework for optimizing peri-implant soft tissue in subcrestally placed implants in single molar cases: integrating transitional and subcrestal zones for biological stability. J Clin Med. (2025) 14:2435. 10.3390/jcm14072435 40217885 PMC11989639

[6] Wang J Cha S Zhao Q Bai D. Methods to assess tooth gingival thickness and diagnose gingival phenotypes: a systematic review. J Esthet Restor Dent. (2022) 34:620–32. 10.1111/jerd.12900 35297167

[7] Samal A Majzoub J Rodriguez Betancourt A Webber L Mazzocco J Wang H High-frequency ultrasound for detecting periodontal inflammation: a preclinical diagnostic accuracy study. J Periodontal Res. (2025). 10.1111/jre.13376 39799460 PMC12371819

[8] Scarfe WC Farman AG White SC Pharoah MJ. Oral radiology—principles and interpretation. St. Louis: Mosby Elsevier (2009) 6:225–43.

[9] Januário AL Barriviera M Duarte WR. Soft tissue cone-beam computed tomography: a novel method for the measurement of gingival tissue and the dimensions of the dentogingival unit. J Esthet Restor Dent. (2008) 20:366–73. 10.1111/j.1708-8240.2008.00210.x 19120781

[10] Sabri H Nava P Hazrati P Alrmali A Galindo-Fernandez P Saleh MHA Comparison of ultrasonography, CBCT, transgingival probing, colour-coded and periodontal probe transparency with histological gingival thickness: a diagnostic accuracy study revisiting thick versus thin gingiva. J Clin Periodontol. (2025) 52:547–60. 10.1111/jcpe.14139 39973090 PMC11949593

[11] Guo X Huang Z Huang J Wei J Li Y Zheng H Accuracy of a cascade network for semi-supervised maxillary sinus detection and sinus cyst classification. Clin Implant Dent Rel Res. (2025) 27:e13431. 10.1111/cid.13431 39898709

[12] Chappuis V Engel O Shahim K Reyes M Katsaros C Buser D. Soft tissue alterations in esthetic postextraction sites: a 3-dimensional analysis. J Dent Res. (2015) 94:187S–93. 10.1177/0022034515592869 26130259

[13] Abdulkarim HH Antoine NM Wang MY Rosales ER Miley DD. Digital assessment of supracrestal tissue attachment and its correlation with dentogingival components. Clin Adv Periodontics. (2024) 14:310–8. 10.1002/cap.10280 38348934 PMC11718352

[14] Razzak MI Naz S Zaib A. Deep learning for medical image processing: overview, challenges and the future. In: Dey N Ashour A Borra S, editors. Lecture Notes in Computational Vision and Biomechanics. Cham: Springer International Publishing (2018). p. 323–50. 10.1007/978-3-319-65981-7_12

[15] Ali M Benfante V Basirinia G Alongi P Sperandeo A Quattrocchi A Applications of artificial intelligence, deep learning, and machine learning to support the analysis of microscopic images of cells and tissues. J Imaging. (2025) 11(2):59. 10.3390/jimaging11020059 39997561 PMC11856378

[16] Schneider L Krasowski A Pitchika V Bombeck L Schwendicke F Büttner M. Assessment of CNNs, transformers, and hybrid architectures in dental image segmentation. J Dent. (2025) 156:105668. 10.1016/j.jdent.2025.105668 40064460

[17] Alsolamy M Nadeem F Azhari AA Ahmed WM. Automated detection, localization, and severity assessment of proximal dental caries from bitewing radiographs using deep learning. Diagnostics. (2025) 15:899. 10.3390/diagnostics15070899 40218248 PMC11988774

[18] Imani M Borda MG Vogrin S Meijering E Aarsland D Duque G. Using deep learning to perform automatic quantitative measurement of masseter and tongue muscles in persons with dementia: cross-sectional study. JMIR Aging. (2025) 8:e63686. 10.2196/63686 40106819 PMC11999904

[19] Yang M Li C Yang W Chen C Chung C-H Tanna N Accurate gingival segmentation from 3D images with artificial intelligence: an animal pilot study. Prog Orthod. (2023) 24:14. 10.1186/s40510-023-00465-4 37121951 PMC10149545

[20] Chen L-C Zhu Y Papandreou G Schroff F Adam H. Encoder-decoder with atrous separable convolution for semantic image segmentation. Proceedings of the European Conference on Computer Vision (ECCV) (2018). p. 801–18

[21] Sandler M Howard A Zhu M Zhmoginov A Chen L-C. Mobilenetv2: inverted residuals and linear bottlenecks. Proceedings of the IEEE Conference on Computer Vision and Pattern Recognition (2018). p. 4510–20

[22] Zhao R Qian B Zhang X Li Y Wei R Liu Y Rethinking dice loss for medical image segmentation. 2020 IEEE International Conference on Data Mining (ICDM) (2020), IEEE. p. 851–60

[23] Gower RM Loizou N Qian X Sailanbayev A Shulgin E Richtárik P. SGD: general analysis and improved rates. International Conference on Machine Learning, PMLR (2019). p. 5200–9

[24] Ronay V Sahrmann P Bindl A Attin T Schmidlin PR. Current status and perspectives of mucogingival soft tissue measurement methods. J Esthet Restor Dent. (2011) 23:146–56. 10.1111/j.1708-8240.2011.00424.x 21649828

[25] Sirinirund B Wang I Ramadan G Kripfgans OD Chan H. Ridge augmentation planning, wound healing evaluation, and peri-implant tissue phenotype assessment with ultrasonography: a case report. Clin Adv Periodontics. (2024) 14:30–7. 10.1002/cap.10234 36700452

[26] Sun S Zhang T Zhao W Gong Z Jia L Gu W Evaluation of palatal mucosal thickness in maxillary posterior teeth using cone-beam computed tomography combined with intraoral scanning: a cross-sectional study on correlating factors. BMC Oral Health. (2025) 25:421. 10.1186/s12903-025-05805-w 40119374 PMC11929230

[27] Li W Xu T Zhang H Lü Y Yang Q Zhu J Digital analysis of periodontal phenotype in the maxillary anterior region. Clin Adv Periodontics. (2025). 10.1002/cap.10353 40123532

[28] Wei L Wu S Huang Z Chen Y Zheng H Wang L. Autologous transplantation tooth guide design based on deep learning. J Oral Maxillofac Surg. (2024) 82:314–24. 10.1016/j.joms.2023.09.014 37832596

[29] Li Y Lyu J Cao X Zhou Y Tan J Liu X. Accuracy of a calibration method based on cone beam computed tomography and intraoral scanner data registration for robot-assisted implant placement: an *in vitro* study. J Prosthet Dent. (2024) 132:1309-e1. 10.1016/j.prosdent.2024.08.009 39245604

[30] Huang Z Zheng H Huang J Yang Y Wu Y Ge L The construction and evaluation of a multi-task convolutional neural network for a cone-beam computed-tomography-based assessment of implant stability. Diagnostics. (2022) 12:2673. 10.3390/diagnostics12112673 36359516 PMC9689694

[31] Ronneberger O Fischer P Brox T. U-Net: convolutional networks for biomedical image segmentation. In: Navab N Hornegger J Wells W Frangi A, editors. Lecture Notes in Computer Science. Cham: Springer International Publishing (2015). p. 234–41. 10.1007/978-3-319-24574-4_28

